# Narrow Alveolar Ridge Management with Modified Ridge Splitting Technique: A Report of 3 Cases

**DOI:** 10.1155/2023/9968053

**Published:** 2023-03-28

**Authors:** Heon-Young Kim, Jung-Hyun Park, Jin-Woo Kim, Sun-Jong Kim

**Affiliations:** Department of Oral and Maxillofacial Surgery, Ewha Womans University Medical Center, Seoul, Republic of Korea

## Abstract

**Purpose:**

In this study, we report the usefulness of implant placement with modified ridge splitting technique from three cases of patients with narrow alveolar ridge.

**Materials and Methods:**

Three patients were those who visited the Department of Oral and Maxillofacial Surgery of Ewha Medical Center for consultation regarding implant placement. Through clinical and radiographic evaluation, narrowed alveolar ridge after tooth loss was confirmed in all three patients. For them, it was necessary to use the modified ridge split technique with bone augmentation for the implant to be well placed with enough bone width.

**Results:**

In all cases, sufficient bone width was confirmed for implant placement, and bone volume was well maintained after prosthetic restoration without any complications. Initial width of alveolar bone was 4.9 mm on average and was well maintained at an average of 7.6  mm at 1-year follow-up after implant installation.

**Conclusion:**

Although the number of subjects in this case report was small and was done by only one surgeon, we suggest that modified ridge splitting technique might be a useful surgical method to enhance narrow edentulous alveolar ridges and enable successful implant placement with shorter healing period compared with single guided bone regeneration.

## 1. Introduction

Osseointegrated implants have been recently used for oral rehabilitation in partially and fully edentulous patients. Successful implant placement requires sufficient bone volume and quality, as well as adequate upper and lower occlusal relationship. Several clinical studies have shown that at least 1 mm of bone width buccal and lingual to the implant surface is needed to assure long-term bone coverage and implant success [1]. However, loss of teeth often leads to a reduced vertical and horizontal alveolar ridge, causing difficulty in implant placement. Various surgical techniques, such as onlay (veneer) block bone grafting with intraoral sources, guided bone regeneration (GBR)/particulate bone grafting, ridge splitting/expansion, and distraction osteogenesis, have been introduced to compensate for narrow alveolar ridges.

The ridge splitting and expansion techniques are surgical methods used to treat horizontal atrophy of the alveolar bone. The main concept of alveolar ridge splitting and expansion is to form a self-space-making defect made of autobone [2]. The ridge splitting technique was first introduced by Tatum in 1986 [3], but was then reintroduced in 1990 by Scipioni et al. [4]. It was renamed the “edentulous ridge expansion (ERE) technique” by Scipioni et al. [5]. In ERE, implant and bone graft materials are placed in intentionally separated bone fragments and enclosed with autogenous bone and periosteum to promote enhanced bone regeneration [6]. In 1994, Summers proposed the ridge expansion technique, which utilizes the viscoelastic properties of bone, applying pressure on the buccal and lingual cortical plate using a Summers's osteotome to increase the width of the alveolar ridge [7]. The effectiveness of ridge splitting and expansion has been demonstrated in a series of clinical, histological, and animal studies [8, 9], and these techniques have been improved with various surgical instruments and equipment, such as chisels, osteotomes, and piezosurgery devices [6, 10–13]. However, all these procedures can cause fracture of the buccal bone plate, receive poor blood supply, and have the possibility of sequestrum formation, so it seemed to need improvement [14].

Herein, we report three cases of patients with a narrow alveolar ridge who underwent implant placement with a modified ridge splitting technique to evaluate the usefulness of this technique. To minimize complications that may occur during ridge splitting and expansion, vertical osteotomy was not performed or was performed only in areas where a large increase in width was required using a microsaw. In addition, ridge preservation was performed simultaneously when extraction of adjacent teeth was necessary.

As a retrospective study, this case report followed the ethical standards according to the World Medical Association Declaration of Helsinki and passed the review by the Institutional Ethics Review Committee of Ewha Womans University Medical Center (Institutional Review Board (IRB) No. SEUMC 2023-01-024).

## 2. Case Report

### 2.1. Case 1

**Figure 1:**
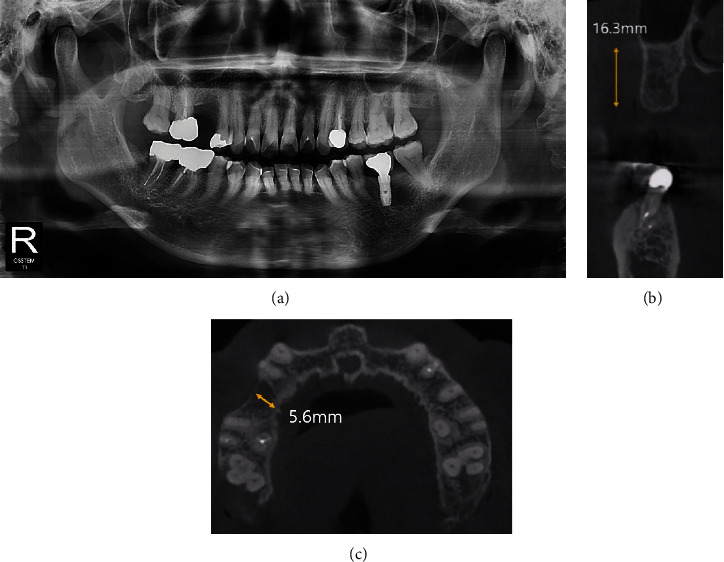
Pre-operative radiographic views (Case 1). (a) Panoramic view. (b) and (c) CBCT view. The height of alveolar bone was sufficient, but it can be seen that the width was somewhat narrowed.

A 60-year-old woman with no underlying conditions other than prescriptions of hormone drugs visited the Department of Oral and Maxillofacial Surgery of Ewha Medical Center for consultation regarding implant placement. The patient's second premolar in the right maxilla had been extracted 8 months ago at a local clinic due to a periapical lesion. Panoramic radiography and cone-beam computed tomography (CBCT) examinations were performed. Radiographic examination revealed a narrowed alveolar ridge at the extraction site, and root caries was suspected under the restoration of the right maxillary first molar. An extraction was planned after further evaluation of this tooth (Figure 1).

**Figure 2:**
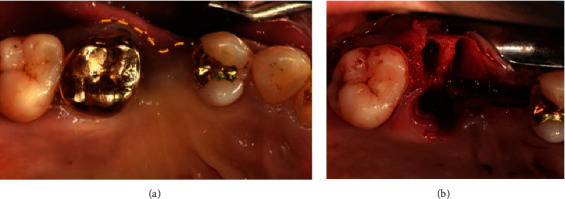
Intraoral views of alveolar ridge split technique with ridge preservation (Case 1). (a) Pre-operative clinical gingiva. (b) Alveolar bone was split and expanded with chisel.

During extraction, alveolar ridge preservation and ridge splitting were performed simultaneously using bone graft materials and absorbable barrier membrane. The patient rinsed with a 0.12% chlorhexidine digluconate solution (Hexamidine, Bukwang, Seoul, Korea) before the operation. Following local anesthesia (2% lidocaine with 1 : 100,000 epinephrine, Yuhan, Korea), a flap including the mid-crestal incision and single vertical incision (mesial area) was reflected to expose the ridge crest. For alveolar ridge splitting (Easy Safe Stable Expanding and Tapping Kit (ESSET Kit)®, Osstem, Korea), vertical (mesial area) and lateral osteotomies were performed on the maxillary alveolar buccal bone plate using a microsaw. Then, the buccal bone plate, on which ridge splitting was performed, was expanded in the buccal aspect using a chisel (Ridge Split Kit®, Osstem, Korea) by 3 ~ 4 mm (Figure 2).

After ridge splitting and expansion, the space between the palatal bone and buccal bone plates and the extraction socket were filled with 0.25 g of mineralized freeze-dried bone allograft (OraGraft®, Lifenet Health, USA), 0.25 g bovine bone mineral (Bio-Oss®, Geistlich Pharm AG, Wolhusen, Switzerland), and absorbable barrier membrane (Bio-Gide®, Geistlich Pharm AG, Wolhusen, Switzerland). In order to achieve a tension-free suture, a periosteal-releasing incision was performed to extend the flap, and after that, the interrupted suture was done.

**Figure 3:**
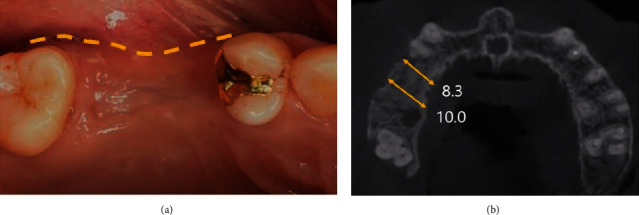
Periodic follow-up at 4 months after operation (Case 1). (a) Intra-oral view. (b) CBCT view. The width of the alveolar bone had increased.

**Figure 4:**
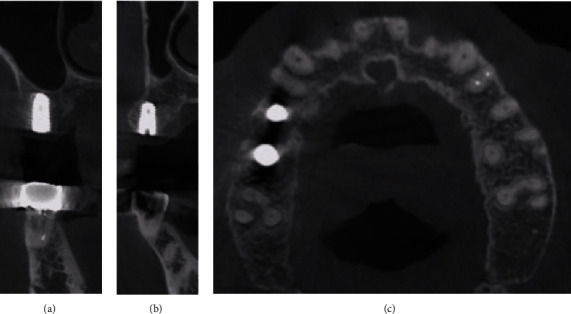
(a–c) Post-implantation radiograph views (Case 1). The width of the alveolar bone was well-maintained, and the implants were placed.

Postoperative instructions were given to the patient. A 7-day supply of antibiotics (250 mg amoxicillin) and analgesics (385 mg ibuprofen) were prescribed along with 0.12% chlorhexidine mouthwash. The sutures were removed 7–10 days after surgery. Periodic follow-up found that the bone graft was stable at 4 months, as observed on radiographs (Figure 3), and implants were subsequently placed (TSIII Sand blasted with alumina and Acid etched surface (SA) 4.0 × 8.5 mm, 5.0 × 10 mm, Osstem, Korea) (Figure 4).

**Figure 5:**
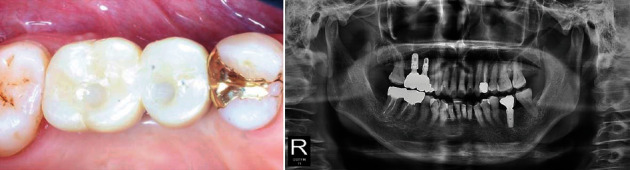
Intraoral and panoramic views after prosthetic rehabilitation (Case 1). The prostheses were maintained well without any specific complications during the follow-up period.

Re-entry surgery was performed 4 months after implant placement. As the implant stability quotient (ISQ) value measured using an Resonance Frequency Analysis (RFA) device was 75–80, suggesting stable implants, prosthetic rehabilitation with full zirconia crowns was fabricated (Figure 5).

### 2.2. Case 2

**Figure 6:**
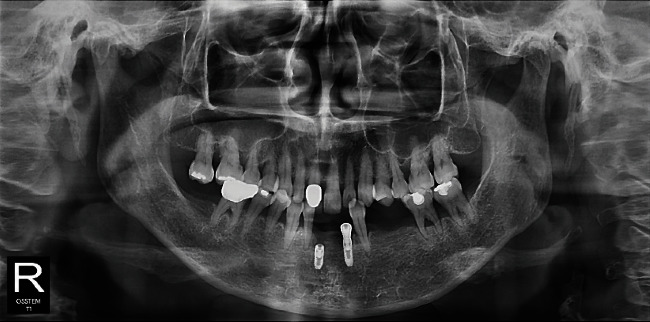
Pre-operative panoramic view (Case 2).

A 63-year-old woman with no systemic specific diseases visited the Department of Oral and Maxillofacial Surgery of Ewha Medical Center complaining of periodontal disease. Panoramic radiography and CBCT examinations were performed. Radiographic examination revealed a narrowed alveolar ridge in the left mandibular posterior area, which required extraction of the left mandibular first molar (Figure 6). The left mandibular first molar was extracted, and the patient received periodontal treatment and management at our hospital for 2 months. Subsequently, implants were placed in the left mandibular first premolar and the left mandibular first molar using the ridge splitting technique.

The patient rinsed with a 0.12% chlorhexidine digluconate solution (Hexamidine, Bukwang, Seoul, Korea) before the operation. Following local anesthesia (2% lidocaine with 1 : 100,000 epinephrine, Yuhan, Korea), a flap including the mid-crestal incision and single vertical incision (mesial area) was reflected to expose the ridge crest. For alveolar ridge splitting (ESSET Kit®, Osstem, Korea), vertical (mesial area) and lateral osteotomies were performed on the mandibular alveolar buccal bone plate using a microsaw. Then, the buccal bone plate, on which ridge splitting was performed, expanded in the buccal aspect using a chisel (Ridge Split Kit®, Osstem, Korea) by 3 ~ 4 mm. The initial length of the osteotomy was prepared to be approximately 3 mm deeper than the desired implant length. After carefully expanding the space between the mandibular buccal and lingual bone plates to prevent fracture of the expanded buccal bone plate, implant drilling was subsequently performed, and 2 implants were placed (TSIII SA 4.5 × 7 mm & 6.0 × 8.5 mm, Osstem, Korea).

**Figure 7:**
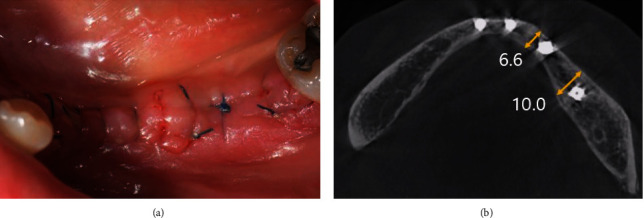
Postoperative intraoral (a) and radiograph views (b) (Case 2). The width of the alveolar bone was well expanded, and we can see that the implant had been placed in the right direction.

After implant placement, a bone graft was performed in the space between the mandibular lingual bone and buccal bone plates. During the procedure, the defects were filled with 0.25-g bovine bone mineral (Bio-Oss®, Geistlich Pharm AG, Wolhusen, Switzerland), and GBR was performed in the horizontal area with absorbable membrane (Bio-Gide®, Geistlich Pharm AG, Wolhusen, Switzerland). In order to achieve a tension-free suture, a periosteal-releasing incision was performed to extend the flap, and after that, the interrupted suture was done (Figure 7). In postoperative panoramic radiograph and CBCT, it was confirmed that implant placement and bone graft with alveolar ridge splitting were well performed.

Postoperative instructions were given to the patient. A 7-day supply of antibiotics (250 mg amoxicillin) and analgesics (385 mg ibuprofen) were prescribed along with 0.12% chlorhexidine mouthwash. The sutures were removed 7–10 days after surgery.

**Figure 8:**
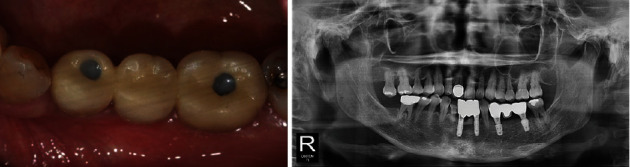
Final prostheses were delivered (Case 2). The prostheses were maintained well without any specific complications during follow-up period.

A follow-up conducted 4 months after the surgery found that the surgical site had healed well without any complications, so re-entry surgery was performed. New high-quality bone had been generated in the surgical site, and the ISQ value using RFA device was measured to be 70, suggesting adequate implant stability. For the final prosthetics, porcelain-fused-to-metal crowns were fabricated (Figure 8).

### 2.3. Case 3

**Figure 9:**
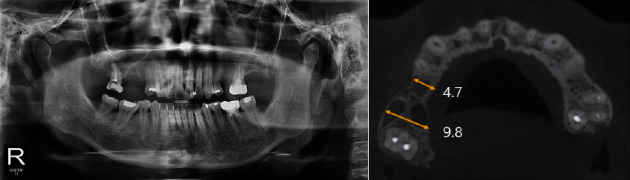
Pre-operative radiographic views (Case 3). In the CBCT view, it can be seen that the width and height of the alveolar bone are atrophied.

A 46-year-old woman without any systemic diseases visited the Department of Oral and Maxillofacial Surgery at Ewha Medical Center for a consultation regarding implant placement. The patient's first molar in the right maxilla was extracted 1 month ago at a local clinic due to a periapical lesion. Panoramic radiography and CBCT examinations were performed. The buccal bone was found to be considerably atrophied by clinical and radiological evaluations (Figure 9). Thus, the patient was scheduled to undergo bone grafting before implant placement.

**Figure 10:**
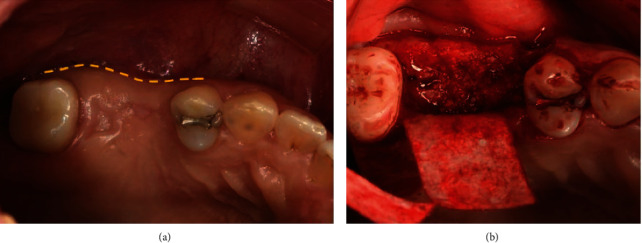
Intraoral views of the alveolar ridge splitting technique with guided bone regeneration (Case 3). (a) Pre-operative site of gingiva. (b) Alveolar bone splitting and expanding with chisel and GBR were done.

**Figure 11:**
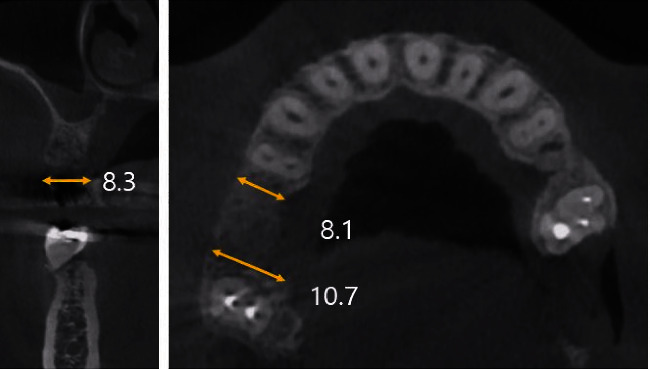
Postoperative CBCT view, showing that the width of the alveolar bone had increased (Case 3).

The patient rinsed with a 0.12% chlorhexidine digluconate solution (Hexamidine, Bukwang, Seoul, Korea) before the operation. Following administration of local anesthesia (2% lidocaine with 1 : 100,000 epinephrine, Yuhan, Korea), a flap including the mid-crestal incision and single vertical incision (mesial area) was reflected to expose the ridge crest. For alveolar ridge splitting (ESSET Kit®, Osstem, Korea), vertical (mesial area) and lateral osteotomies were performed on the maxillary alveolar buccal bone plate using a microsaw. Then, the buccal bone plate on which ridge splitting was performed was expanded in the buccal aspect using a chisel (Ridge Split Kit®, Osstem, Korea) by 3 ~ 4 mm. After ridge splitting and expansion, the space between the palatal bone and buccal bone plates and the extraction socket were filled with 0.25 g bovine bone mineral (Bio-Oss®, Geistlich Pharm AG, Wolhusen, Switzerland) and absorbable barrier membrane (Bio-Gide®, Geistlich Pharm AG, Wolhusen, Switzerland). In order to achieve a tension-free suture, a periosteal-releasing incision was performed to extent the flap, and after that, the interrupted suture was done (Figures 10 and 11).

**Figure 12:**
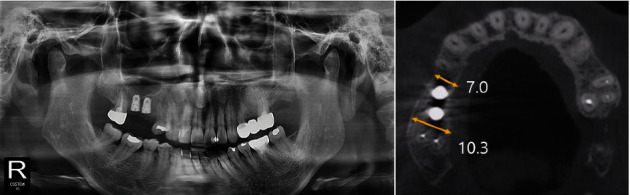
Post-implantation radiograph views (Case 3). The width of the alveolar bone was well-maintained, and the implant was placed.

Postoperative instructions were given to the patient. A 7-day supply of antibiotics (250 mg amoxicillin) and analgesics (385 mg ibuprofen) were prescribed along with 0.12% chlorhexidine mouthwash. The sutures were removed 7–10 days after surgery. After periodic follow-up, bone graft was found to be stable at 7 months, as observed on radiographs, and implants were subsequently placed (TSIII SA 4.5 × 8.5 mm, Osstem, Korea) (Figure 12).

Re-entry surgery was performed 4 months after implant placement. As the ISQ measured using an RFA device was 84–85, suggesting adequate implant stability, prosthetic rehabilitation with full zirconia crowns was fabricated.

## 3. Results

In all three cases, sufficient bone width was secured for implant placement without complications, and bone volume was well-maintained after prosthetic restoration. In the three patients, the width of alveolar bone was 4.9 ± 0.5 mm on average and 8.3 ± 1.1 mm immediately after surgery, whereas the width of alveolar bone during the implant placement and 1-year follow-up period was 7.6 ± 1.0 mm (Table 1).

## 4. Discussion

Rehabilitation of partially or totally edentulous patients with dental implants has become a common treatment and yields reliable long-term results. To achieve successful implant therapy, availability of adequate amounts of bone in terms of horizontal as well as vertical dimensions is the first requirement, but local conditions of edentulous alveolar ridges may be unfavorable for implant placement [15].

Previous studies have investigated the resorption patterns of alveolar bone after tooth loss or extraction due to trauma, periodontal disease, and birth defects. The greatest amount of bone loss usually occurs in the horizontal dimension, mainly on the facial side of the ridge. There may also be loss of vertical ridge height, which has been reported to be the most pronounced on the buccal aspect [16–18]. This resorption process results in a narrower and shorter ridge, and the effect of this resorption pattern is the relocation of the ridge to a more palatal/lingual position [19]. The size of the residual ridge is reduced most rapidly in the first 6 months, when approximately 0.90–3.6 mm of buccal bone and 0.4–3.0 mm of lingual bone are absorbed [20, 21], but bone resorption activity in the residual ridge continues throughout life at a slower rate, resulting in the removal of large amounts of jaw structure [22]. As the amounts and rates of alveolar ridge reduction after tooth loss vary, several surgical methods may be applied for implant placement in the edentulous space.

Among the various surgical techniques for implants on the atrophic alveolar bone, the ridge splitting and expansion technique is considered one of the most successful horizontal bone augmentation procedures. This approach was first introduced by Tatum in 1986 [3] but was then reintroduced in 1990 by Scipioni et al. [4]. The method involves the splitting of the vestibular and buccal cortical plates [23, 24] and further expanding the gap with Summers's osteotomes [23–25]. A minimum of 3 mm of bone width, including at least 1 mm of cancellous bone, is required to place an osteotome between cortical plates and expand the cortical bony plates.

In many studies, such as those by Scipioni et al. [4] and Summers [23, 24], this technique was used on narrow ridges to obtain successful results [4, 23, 24]. Scipioni and Bruschi performed a ridge expansion technique that included a split-thickness flap and bone-releasing incision to immediately place the implants, and did not graft any materials in the gap. In a total of 170 patients with 329 implant placements, the 5-year implant survival rate was reported to be 98.8%. Summers invented the round osteotome for placement of cylinder-shaped implants. This osteotome greatly increased the success rate of implant placement, with failure noted in only 5 of 143 implants in the maxilla. In addition, according to a paper published by Sethi and Kaus, 449 implants were placed using ridge split technique in 150 patients, and success rate was reported as 97% in 27-month follow-up [26]. Similarly, in a paper by Ferrigno et al., 97.6% success rate was reported in 40 patients when a total of 82 implants were placed using the same technique [27]. Along with the change in the technique, there was also a change in the instrument. In the case of an existing chisel, it is difficult to control the splitting in hard bone quality, and in the case of a saw, there is a disadvantage that is can damage lips or tongue. Therefore, a method using a microsaw or an ultrasound device in a bone width of 2 mm or hard bone quality was introduced [28, 29].

As such, although other instruments may be developed and surgical techniques may change, the ridge splitting technique should be considered a predictive and advantageous technique. In addition, since implants were placed between the buccal and cortical bones where bone grafts were carried out, the blood supply between plates is sufficient [30]. This is similar to the healing that occurs after tooth extraction, resulting in the generation of qualified new bone, which facilitates the osseointegration of the implant [31]. Furthermore, alveolar rige splitting has the advantage of reducing the overall surgical time because it is possible to increase the width of alveolar bone and place the implant simultaneously in a single surgery. Moreover, the bone generated in this operation has a low absorption rate, resulting in a high long-term success rate. However, there is still controversy over whether bone graft material should be placed into the gap or not. Kolerman et al. conducted a study on the long-term outcomes of ridge expansion using the osteotome procedure followed by implants in combination with GBR in patients with atrophic maxillary alveolar ridges and reported significant increases in the ridge width over the study period (pre-op ridge width values increased significantly from 3.73 ± 0.67mm to 7.19 ± 0.80 mm) [32]. In 2014, Ella et al. investigated 32 patients with 64 implants, where 17 patients had synthetic bone grafts between gaps after ridge expansion, and 15 patients did not. When comparing the frequency and amount of bone resorption after 6 months of follow-up, it was observed that the non-graft group showed more resorption and higher frequency [33]. As already stated, the ridge splitting technique is a predictive technique with many advantages. However, there are also limitations to its use, including the need for proper cortical bone thickness and cancellous bone to prevent fractures of the buccal bone, difficulty in applying it to aesthetic areas due to inclined implant implantation, and the need for careful surgical skill. In addition, it does not compensate for vertical bone loss. Many studies recommend ridge splitting/expansion for alveolar ridge with a width of at least 3 mm [34]. However, in clinical practice, absorption progresses rapidly, and implants are often needed in alveolar ridges with a width of less than 3 mm. In these cases, GBR can be performed to repair peripheral defects, such as fractures of the buccal bone plate, vertical alveolar bone defects, and dehiscence defects. In patients with uncertain prognosis, GBR can be performed first with ridge splitting/expansion, followed by implant placement. Additionally, the choice of method will depend on whether the implants to be placed have primary stability in the expanded ridge or not [34]. However, in contrast to the above-mentioned articles, Coatoam and Mariotti recommended bone grafting in the space between bone plates without placing implants in case of incorrect fixation. Additionally, in this paper, when the stability of the bone plate was questionable despite correct implant placement, the bone plate was ligated and fixed with wires [6]. Furthermore, if postoperative complications or bone loss occur, this may cause larger bone defects before treatment, so careful surgical process and postoperative care are required.

In our cases, in order to minimize complications that may occur during ridge splitting and expansion, vertical osteotomy was not performed or was performed only in areas where a large increase in width was required using a microsaw. In addition, a difference from previous papers could be seen in that ridge splitting & expansion and ridge preservation were performed simultaneously when extraction of adjacent teeth was necessary. As such, the surgical method selected considered various factors, and the surgeries were completed successfully without any special complications.

## 5. Conclusion

Although the number of subjects in this case report was small and there was only one surgeon, we can conclude that the modified ridge splitting technique might be a useful surgical method to enhance narrow edentulous alveolar ridges, enabling successful implant placement with a shorter healing period compared to a single GBR. In addition, if accurate diagnosis of alveolar bone defects, patient selection suitable for indication, and careful surgery are combined, predictive results can be achieved.

## Figures and Tables

**Table 1:** Comparison of alveolar bone widths before and after surgery. There was some resorption, but as a result, it can be seen that the width of the alveolar bone increased after ridge splitting and expansion technique.

Case number	Pre-M	Pre-D	Pos-M	Pos-D	Pi-M	Pi-D
Case 1	5.6	8.3	10	12.3	9.1	11.8
Case 2	4.4	10.4	7	11	6.6	10.8
Case 3	4.7	9.8	8.1	10.7	7.0	10.3

Pre-M: pre-op mesial, Pre-D: pre-op distal, Pos-M: post-op mesial, Pos-D: post-op distal, Pi-M: post-implantation mesial, Pi-D: post-implantation distal.

## Data Availability

Data supporting this research article are available from the corresponding author or first author on reasonable request.
